# A Review of Different Stunning Methods for Poultry—Animal Welfare Aspects (Stunning Methods for Poultry)

**DOI:** 10.3390/ani5040407

**Published:** 2015-11-30

**Authors:** Charlotte Berg, Mohan Raj

**Affiliations:** 1 Department of Animal Environment and Health, Swedish University of Agricultural Sciences, POB 234, Skara SE-532 23, Sweden; 2 Visiting Fellow, School of Veterinary Sciences, University of Bristol, Langford BS40 5DU, UK; E-Mail: M.Raj@bristol.ac.uk

**Keywords:** broiler, laying hen, slaughter, stunning, turkey, welfare

## Abstract

**Simple Summary:**

To avoid unnecessary suffering, poultry and other animals are often made unconscious, *i.e.*, stunned, prior to exsanguination at slaughter. This review describes various stunning methods used for the commercial slaughter of poultry, their mode of action and also the main animal welfare aspects. Furthermore, it includes a short discussion on possible future development of new methods in the field of poultry stunning.

**Abstract:**

Electrical water bath stunning is the most commonly used method for poultry stunning prior to slaughter, but has been questioned on animal welfare and product quality grounds. Controlled atmosphere stunning (CAS) methods, involving a variety of gas mixtures, have become increasingly common, at least in Europe. CAS methods have been perceived as an improvement from an animal welfare perspective, partly because birds can be stunned without prior shackling, and are generally considered to result in improved product quality compared to water bath stunning. However, there would still be an interest in alternative stunning methods especially for small to medium size poultry slaughterhouses. This review presents an overview of the modes of action and the technical aspects of poultry stunning methods, including novel and emerging stunning technologies.

## 1. Introduction

The stunning of animals prior to slaughter is practiced throughout the world. The requirement for stunning, *i.e.*, inducing unconsciousness, prior to slaughter (neck cutting) is based on the understanding that (a) animals are sentient beings and (b) neck cutting causes pain and suffering, which can be avoided by pre-slaughter stunning. Hence, most guidelines and regulations related to animal welfare at slaughter include a list of known stunning methods applicable to different species of animals and certain minimum standards for each method required to induce immediate unconsciousness that lasts until death occurs by bleed-out [1,2]. The physiological bases of stunning methods and associated parameters have been thoroughly reviewed, for example by the European Food Safety Authority (EFSA) [3]. Scientific studies carried out under controlled laboratory conditions involve the recording of the spontaneous and evoked electrical activity in the brain using electroencephalograms (EEGs) to ascertain the depth and duration of unconsciousness induced by a stunning method. In general, unconsciousness is assessed by criteria such as epileptiform activity, profoundly suppressed or quiescent EEGs, and/or abolition of evoked electrical activity in the brain (e.g., somatosensory evoked potentials) depending upon the stunning method. EFSA has recently published guidance on the assessment criteria for studies evaluating the effectiveness of stunning methods regarding animal protection at the time of killing [4].

In Europe, the welfare of animals, including poultry, at the time of killing is protected under Regulation (EC) 1099/2009 [1]. Stunning methods currently permitted in this Regulation for use in poultry slaughterhouses within the EU are penetrating and non-penetrating captive bolt, firearms, head-only electrical stunning, head-to-body electrical stunning, electrical water bath, carbon dioxide in two phases, carbon dioxide associated with inert gases, and inert gases only. Individual member states may have chosen the possibility to retain national legislation, if it was in place at the time of the adoption of the Regulation in 2009 and offers greater protection through higher standards of welfare at slaughter. For example, Sweden requires a minimum current of 120 mA not only for higher frequencies but also for birds stunned in low frequency electrical water baths [5], whereas the EU Regulation 1099/2009 only requires 100 mA for frequencies up to 200 Hz. The methods applied commercially in the EU are electrical waterbath stunning (81% of broilers) and gas stunning (19%), with the mechanical stunning methods not typically used [6]. It is generally acknowledged that the existing methods may be refined or replaced in order to improve animal welfare and/or meat quality and EU Regulation 1099/2009 specifically encourages innovation to achieve higher standards. EFSA guidance on the assessment criteria for studies evaluating stunning methods is also intended for facilitating such innovations.

This review, based on a keynote lecture [7], deals with the mode of action of existing or potential stunning methods for poultry, with an emphasis on animal welfare but also taking other aspects into account. This review is written based on current European practices and both legislation and slaughterhouse practices may be different in other regions. However, we would like to emphasize that the physiological reactions of the birds to various stunning interventions are universal, and hence globally relevant.

## 2. Stunning Methods

### 2.1. Electrical Waterbath Stunning

**Figure 1:**
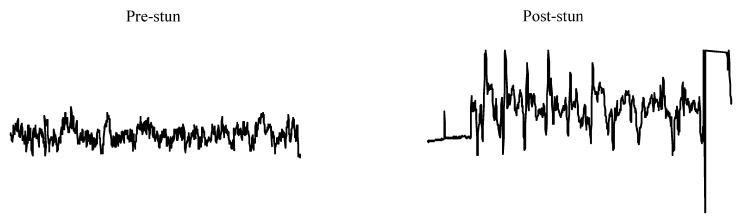
Electrical stunning-induced 3–4 Hz polyspike activity in the electroencephalogram (10 s epochs) of a chicken.

**Figure 2:**
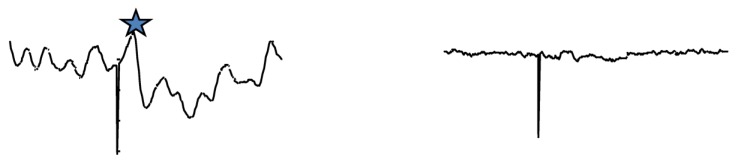
Electrical stunning-induced abolition of somatosensory potentials in the brain of a chicken (Vertical line corresponds to the stimulus, the star indicates the response).

In general, effective electrical stunning involves stimulation of the brain with a current of sufficient magnitude to induce generalized epilepsy characterized by the occurrence of highly synchronized 8–13 Hz activity in the EEG, which is deemed to be incompatible with the persistence of consciousness and sensibility [8]. However, research carried out during the 1980 on electrical water bath stunning of chickens suggested that, unlike red meat species, only a small proportion of birds developed epileptiform activity in the brain [9,10,11,12] and about 90% of birds that develop epileptiform activity showed low frequency (<3 Hz) polyspike or spike and wave activity [13] (Figure 1). This observation was thought to be disconcerting on bird welfare grounds [13,14] because literature concerning epilepsy in humans suggested that the manifestation of spike-wave discharges or generalized spikes of 3–4 Hz in the EEG is not always associated with unconsciousness. Owing to this uncertainty, further research carried out during the 1980s in Germany and the UK was very much focused on determining minimum currents necessary to induce cardiac ventricular fibrillation at stunning [15] or abolition of evoked electrical activity in the brain. In the latter case, the abolition of such activity was tested using somatosensory evoked potentials induced by electrical stimulation of the radial nerve [10] (Figure 2). Induction of cardiac ventricular fibrillation at stunning will lead to the immediate death of the stunned bird and therefore eliminate the risk of recovery of consciousness following stunning. This can be achieved in a majority of birds by delivering 120 mA per bird in the water bath supplied with a 50 Hz sine wave alternating current (AC). This combination of electrical stunning parameters also leads to the onset of a quiescent EEG, which is also associated with abolition of evoked electrical activity in the brain. However, induction of cardiac arrest without the induction of epileptiform activity indicative of unconsciousness is controversial on bird welfare and ethical grounds.

**Figure 3:**
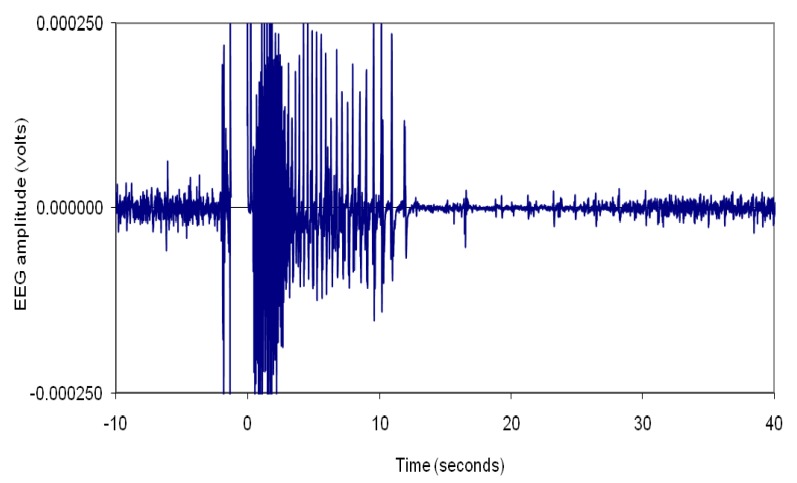
Electrical stunning-induced 8–13 Hz epileptiform activity in the electroencephalogram of a chicken. The pre-stun EEG activity is presented between −10 and 0 s. The spike pattern occurring just prior to 0 is an artifact.

Nevertheless, further research carried out in the UK demonstrated that, as in red meat species, effective electrical stunning of chickens (head-only or water bath stunning) indeed leads to epileptiform EEG activity known to be associated with unconsciousness, *i.e.*, highly synchronised 8–13 Hz activity in the EEG [16,17,18,19,20] (Figure 3). These studies also revealed that the induction of epileptiform activity in the brain of chickens is dependent on the waveform and frequency of the current used in the water baths, which is the scientific rationale for the minimum currents stipulated for different frequency ranges in the EU Regulation 1099/2009. The results of these studies also indicated that sine wave alternating current (AC) is more effective than pulsed direct current (pDC) in terms of inducing epileptiform activity in the brain of chickens (see review by Raj [21]).

Although Raj [14] speculated, based on the available scientific evidence at that time, that chicken brain anatomy and physiology could be the reason for differences between mammals and birds, it is now evident that the discrepancy between the studies is probably due to differences in the methodology used for recording EEG data, *i.e.*, low data sampling rates and analogue EEG recording systems with limited frequency bands were used in early studies whereas high data sampling rates and digital EEG recording systems with wider frequency bands were used in the latter studies. It is worth mentioning that induction of epileptiform activity in the brain following the electrical stunning of animals, including poultry, is now a requirement under EU Regulation 1099/2009, and therefore, any new electrical stunning method or modification to the existing method must clearly demonstrate that epileptiform activity is induced in the brain. This knowledge has not previously been widely disseminated or understood, which is not conducive to achieving higher standards of welfare at slaughter.

Another point of contention is that a stunning method should induce immediate loss of consciousness. Previous EFSA reports [3,22] used a time frame of “within a second” as a measurable time limit under practical conditions to be considered as “immediate”. However, it could be argued that exposure to gas mixtures (gas stunning) does not induce immediate loss of consciousness (*i.e.*, within a second) and why should electrical water bath stunning of poultry abide by this standard? A simple answer to this question would be that birds will suffer severe pain and distress if electrical stunning is not immediate [23].

There are bird welfare issues not directly related to the electrical parameters, but to the design of the water bath stunner itself and the process of handling the birds before they are stunned. Birds have pain receptors in their legs [24] and, hanging live and conscious birds upside down by their legs in a metal shackle, which is a pre-requisite for water bath stunning, is often mentioned as one of the major welfare problems in relation to water bath stunning [25,26]. Distress and pain during shackling prior to stunning can be affected by shackle design, time from shackling to stunning, and the design of the entrance to the water-bath, where poor design can lead to painful pre-stun electric shocks. It is known that, under the multiple bird water bath stunning system, all the birds passing through the electrified water bath will be exposed to a constant voltage, and the electrical flow through each bird will be dependent on impedance in the pathway of each bird [25,27]. This means that if a flock, for example, is of uneven size and weight, different birds will receive different amounts of current, and some birds may not receive enough to induce generalized epilepsy in the brain and some others may receive more current than necessary to induce epileptiform activity, hence suffering carcass and meat quality defects. Owing to this, multiple bird water baths are not conducive to achieving effective stunning or acceptable meat quality in all the birds [28].

The problems associated with electrical water bath stunning are further confounded by the fact that a plethora of current levels (Amp), waveforms (sine wave AC or pulsed DC), and frequencies (Hz) are used commercially [28,29]. The relationship between these different variables is complex, which is reflected by the fact that legislation often requires a minimum current level that is directly linked to the frequency applied, as it has been shown that higher frequencies (*i.e.*, shorter wavelengths) require higher amperage to achieve an effective stun [1,28]. In addition, it has been questioned whether the highest frequencies will result in effective stunning at all, although birds may still appear paralyzed and it may hence be very difficult to identify poorly stunned birds in the commercial slaughterhouse situation. Similar reasoning applies to pulsed DC stunning of poultry, where the pulse width is considered a crucial factor [20]. However, electrical water bath stunning will most likely be continued within the foreseeable future for economic and commercial feasibility reasons [6], although EFSA [23] has recommended that alternatives should be developed/implemented, as the complexity of multiple-bird electrical water bath stunning systems used in poultry slaughterhouses is not conductive to maintaining good animal welfare. It is generally agreed that electric water bath stunning may result in poor product quality, mainly related to severe muscle contractions resulting in petechial blood splash and fractures [30,31], more so at higher currents. This is not considered an animal welfare problem as birds are rendered unconscious, but if such problems result in staff turning down the current settings to improve meat quality this may certainly be a risk for impaired stun quality and thereby poor bird welfare.

### 2.2. Individual Electrical Stunning of Poultry

#### 2.2.1. Head-Only Electrical Stunning

**Figure 4:**
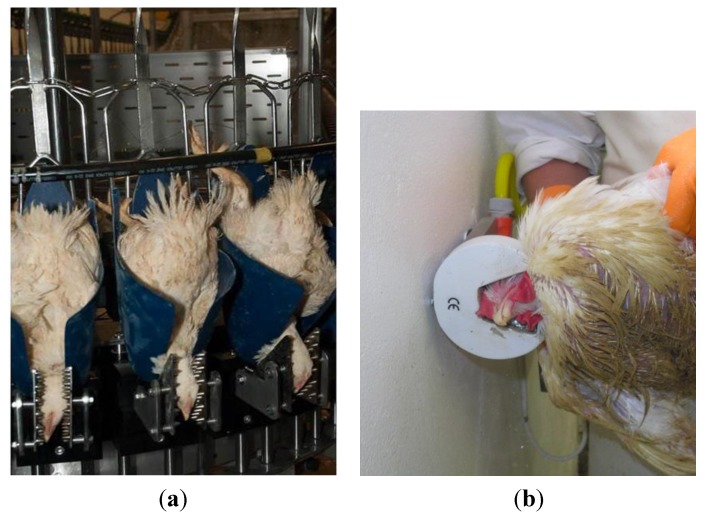
(**a**) Head-only electrical stunning of chickens using dry electrodes, large-scale system. Photo: B Lambooij; (**b**) Head-only electrical stunning of chickens using dry electrodes, small-scale system.

There are systems commercially available for head-only electrical stunning of poultry using dry electrodes. In this system, birds are restrained in a cone and two electrodes are placed on either side of the head of each bird (Figure 4a) [32]. After stunning, the birds are automatically transferred to a shackle line moving in synchrony with the cones and carried for slaughter and processing. The advantages of this system include application of a constant current to individual birds and electronic recording of the current profiles. These could be used by the veterinary control officials for auditing/verification purposes, as required by EU Regulation 1099/2009. Birds which are not or only partially stunned (e.g., due to poor electrical contact) are identified by an LED light that runs synchronously with each bird. This facilitates the machine operator to take action by removing the bird from the shackle or applying a back-up stunning instrument. There are also less automated systems for small-scale slaughter, where the head of the bird is placed manually in contact with the two electrodes (Figure 4b).

#### 2.2.2. Head-to-Cloaca Electrical Stunning

**Figure 5:**
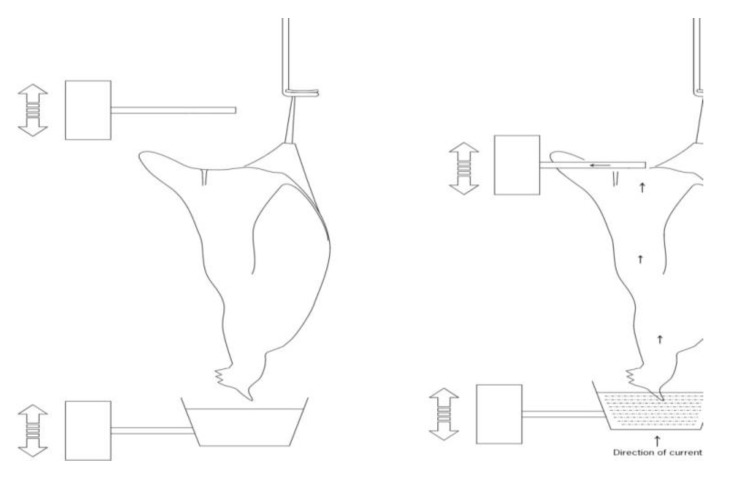
Head-to-cloaca electrical stunning of chickens. Illustration: B. Lambooij.

As a result of the animal welfare disadvantages of the conventional multiple bird electrical water bath stunning system, a novel method of head-to-cloaca electrical stunning of individual birds has been developed in the Netherlands (Figure 5). The bird is inverted and placed in a water-bath, which constitutes one of the two electrodes necessary. Another electrode is then automatically placed on the cloaca of the birds, and thereby the electric circuit is closed [33]. Such a system not only stuns the birds, but also induces cardiac arrest when supplied with appropriate electrical parameters, which has several bird welfare advantages [33]. The system has been shown to result in better product quality (less blood splash) as compared to conventional water-bath stunning [34]. However, the problem of having to shackle conscious poultry still remains. The system would be relevant for medium or large size slaughterhouses but has not yet been fully commercialized [6].

### 2.3. Gas Stunning

Gas stunning is often referred to as “Controlled Atmosphere Stunning” or CAS systems. Such systems have become increasingly common during the last 20 years, mainly as a result of the animal welfare and product quality advantages in comparison with the water bath stunning. Gas stunning does not lead to immediate unconsciousness, but rather induces unconsciousness gradually.

Several different gas combinations are used commercially. When carbon dioxide only is used, the gas is administered in two phases. Birds are first exposed to relatively low concentrations (<40% by volume in air), and then, once the birds are unconscious, they are exposed to a higher concentration (approximately 80%–90% by volume in air). Both mammals and birds have chemoreceptors sensitive to carbon dioxide, and birds will react by e.g., headshaking or gasping already at relatively low concentrations [35,36]. It has been hypothesized that birds will experience pain and distress if exposed to carbon dioxide at high concentrations (e.g., >40% by volume) [21]. Therefore, a two-phase system is considered to minimize the discomfort caused by contact with an aversive gas in high concentrations [1], and the multi-stage systems currently commercially available can be seen as a further development of this concept [37,38]. Nevertheless, exposure to high concentrations in the second phase ensures unconsciousness lasts long enough, *i.e.* until brain death occurs in birds through bleed out.

Carbon dioxide can also be used in combination with inert gases, *i.e.*, argon, nitrogen or both.

Inert gases, such as argon or nitrogen, have anesthetic properties but only under hyperbaric conditions. Exposure to inert gases leads to progressive hypoxia in the birds. Neither mammals nor birds have chemoreceptors for inert gases, and they do not experience aversion when coming in contact with such gases, which is the reason why inert gases have been recommended in some instances as preferable from an animal welfare point of view. However, later studies have indicated that birds stunned using inert gases may still experience negative effects, such as vigorous wing flapping and convulsions, as a result of the hypoxia. Some researchers have argued that although the respiratory discomfort caused by inhalation of carbon dioxide is unpleasant, it may still be preferable to the risk of vigorous wing flapping and associated trauma seen when inert gases are used to create hypoxia [39].

From an animal welfare perspective, gas stunning has several advantages over water bath stunning. One of the major advantages is the fact that with gas stunning, there is no need for inverting and shackling conscious birds, as the birds are instead brought into the stunner either sitting on a conveyor belt or when remaining in their transport crates [37]. Based on animal welfare aspects the latter is preferred, as this also avoids the possible welfare issues linked to emptying the containers onto the conveyor belt. This absence of live shackling is also perceived as positive from a working environment point of view [6]. Furthermore, gas stunning is less sensitive to variations in bird size and conformation than water-bath stunning. From a product quality perspective, gas stunning is generally considered superior to water-bath stunning, and has substantial capacity in terms of number of birds stunned per hour. However, the gas stunners currently available on the market are rather large and involve relatively high investment costs [6], and they can only be used in combination with a certain transport container system. Hence the system involves large investments and is therefore mainly used at large-scale slaughterhouses. There have also been discussions about public perception of the concept of stunning or killing birds using gas; as such the method has historic connotations.

### 2.4. Low Atmosphere Pressure System (LAPS)

The LAPS operates by removing air from a sealed chamber, in which transport containers full of birds have been placed. The birds are rendered unconscious by a gradual reduction of oxygen tension in the chamber, leading to progressive hypoxia [40]. Hence, the induction of unconsciousness in this system is, as is the case also for gas stunning, not instantaneous. The LAPS would share several advantages with the gas stunning systems, e.g., the possibility to stun birds directly in their transport containers, eliminating live bird handling and shackling of conscious birds, provided this novel method is also clearly demonstrated to be humane. The LAPS is approved by the USDA for commercial use in the US [41] and is used in one poultry slaughterhouse there [6], but is currently not permitted in the EU [1]. The system would mainly be relevant for medium or large-size slaughterhouses [6].

Following a request from the European Commission, the EFSA Panel on Animal Health and Welfare was recently asked to deliver a scientific opinion on the bird welfare aspects of such systems. A limited number of scientific publications on the LAPS are currently available, and not all of these have evaluated bird welfare aspects. When working on mandates related to new stunning systems, the EFSA working group is expected to follow the published guidance on the assessment of studies evaluating the effectiveness of stunning interventions regarding animal protection at the time of killing [4]. This evaluation showed that based on the papers submitted, it was not possible to characterize the stunning intervention in a consistent way with respect to key parameters and their impact on the welfare of the chickens, including the variability between birds in relation to the time to onset of unconsciousness based on EEG criteria. It further showed that the estimates of time to loss of consciousness based on behavioral criteria were not fully supported by the results from the studies presented to the Panel. The EFSA panel concluded that it was unclear from these publications whether the rate of decompression used in the LAPS system induces unconsciousness and death without causing avoidable pain and suffering [42]. This means that more research is necessary before a full assessment of the system can be made, if it is to be considered for a future inclusion in the EU legislation. In this regard, the EFSA guidance document provides relevant criteria [4].

## 3. Future Development

In addition to refinements and improvements of the methods mentioned above, it is also possible to foresee the development of new stunning techniques provided that they can be proven to be acceptable from an animal welfare point of view. This means that they should ensure a level of welfare at least equivalent to that ensured by the existing methods permitted by law in the EU, and not be prone to manipulation for meat quality or financial reasons. Furthermore, to be commercially relevant such methods would need to be economically viable and safe from a worker’s health perspective. Two potential new stunning methods, transcranial magnetic stimulation and the use of microwave energy, have been reported in the scientific literature.

Transcranial magnetic stimulation (TMS) of the brain is a non-invasive potential method for the stunning of broilers and other types of poultry. To apply electromagnetic induction, *i.e.* create an intense magnetic field, a TMS probe containing a copper coil can be placed close to the skull of the bird, and an electric current charged by a generator induces the magnetic stimulus within the brain cortex surface [43]. Although it does not produce general epileptiform activity, this procedure can result in behavioral signs and an appearance of the EEG characteristic of unconsciousness. According to this, the method may have the potential for a future development into a short-lasting, reversible stunning method [43]. However, it is currently only at an experimental stage.

Using microwaves is another possible method of stunning not only for poultry but also for larger animals. Such microwaves involve the use of frequencies between 300 MHz and 300 GHz [44], which lead to an increased temperature of the brain. For stunning purposes, the aim is to achieve a brain temperature at which hyperthermic syncope would occur, *i.e.*, between 43 °C and 50 °C [45]. One advantage of using this method for stunning prior to slaughter would be that controlled irradiation can induce a reversible stun, when the energy is applied in such a manner that the bird is rendered unconscious, without tissue damage. This method is of potential interest in relation to religious slaughter [44], but also for conventional slaughter. The microwave method, which must not be confused with standard household microwave oven technology, is still at the experimental stage and research has so far mainly been focused on mammals, even if the results should be applicable also to poultry.

The possibility of head-only electrical stunning of poultry prior to shackling and neck cutting, as carried out in rabbits, should not be excluded. In this regard, rabbits are taken out of their transport crate individually, stunned head-only using a pair of stunning tongs, shackled and bled out by a single operator.

Possibilities of further developing different gas stunning methods are also discussed within the scientific community and the poultry slaughterhouse industry, including equipment better suited for small- or medium-sized slaughterhouses. Furthermore, gas combinations other than the ones currently prevailing may be an option, as is the development of other methods of distributing the gas to the birds, such as systems involving gas-filled foam.

## 4. Conclusions

In summary, it can be concluded that electrical water bath stunning is still the most commonly used method for poultry prior to commercial slaughter but controlled atmosphere stunning methods are becoming increasingly common, especially at larger slaughterhouses. All currently available stunning methods have their advantages and disadvantages, in relation to e.g., animal welfare, product quality and costs. When new stunning methods are developed and introduced, it is crucial to ensure that these technologies can provide a reliable and profound stun quality and that the brain mechanisms associated with the induction and maintenance of unconsciousness are adequately investigated and reported, to avoid unnecessary suffering in the birds.
